# Force-differential steering of a track-based robotic colonoscope: experimental evaluation in single and multi-bend pipes

**DOI:** 10.3389/frobt.2026.1904532

**Published:** 2026-09-09

**Authors:** Jiayang Du, Lin Cao, Sanja Dogramazi

**Affiliations:** School of Electrical and Electronic Engineering, The University of Sheffield, Sheffield, United Kingdom

**Keywords:** colonoscopy, force-differential steering, robotic colonsocpe robotic endoscope, self-propelling, track-based

## Abstract

This work presents a laboratory-scale track-based robotic colonoscope incorporating a force-differential steering mechanism. A syringe-driven hydraulic system selectively retracts individual tracks to generate asymmetric track-wall contact forces for steering. A theoretical model was developed to describe the relationships between hydraulic input, normal-force modulation, and steering torque and to provide a first-order framework for the mechanism. The prototype, was tested with force sensing and an IMU, was evaluated for straight propulsion and steering in single- and multi-bend flexible pipes. Hydraulic actuation tests showed a near-linear increase in track-wall normal force with increasing syringe input, although the measured force was substantially lower than the theoretical prediction. Steering experiments showed that motion without active steering produced approximately 35° of yaw before traction loss, whereas force-differential steering increased the yaw angle to approximately 45° and enabled negotiation of a 120° bend. Tests under different friction conditions further showed that reduced wall friction impaired steering performance. In a continuous S-shaped pipe, two of three independent trials successfully completed both bends, while one trial exhibited incomplete yaw recovery, indicating trial-to-trial variability and sensitivity to traction conditions. Overall, these results provide proof-of-concept evidence for force-differential steering in a tracked robotic colonoscope and demonstrate that straight propulsion and active planar steering can be achieved within a common tracked locomotion architecture. Further miniaturisation, improved hydraulic control, and evaluation in more physiologically representative environments are required before clinical applicability can be assessed.

## 1 Introduction

Stable navigation in the highly curved, compliant, and slippery colon cannot rely on propulsion alone, as insufficient steering control causes deviation from the centreline, unstable wall contact, and failure to reorient in bends (Santucci and Velez, 2024), which increases mucosal stress and prolongs the procedure (Eickhoff et al., 2010) (Alazmani et al., 2016). Active steering therefore plays a crucial role, and prior work has explored multiple strategies, including differential, articulated, magnetic assisted, and compliant continuum based approaches. Differential steering offers simplicity and fast response in non-medical pipe locomotion environments, but can be less efficient in high friction or small radius pipes (Roh and Choi, 2005; Oya et al., 2005; Elankavi et al., 2022). In the context of endoscopy, articulated steering can provide accurate orientation but is also limited by structural complexity and size constraints (Vucelic et al., 2006). Magnetic assisted steering enables wireless control but depends on large external magnets and suffers rapid force decay with distance (Wang et al., 2008) (Verra et al., 2020). Compliant or continuum steering achieves smooth bending through pressurized chambers, though slow response and deformation coupled actuation reduce its precision (Dehghani et al., 2017) (Chen et al., 2022) (Giri et al., 2025).

Roh et al. (Roh and Choi, 2005) presented a differential drive in pipe robot controlled by multiple motors, yet its structurally complex multi-actuator configuration resulted in a bulky form difficult to scale down and therefore unsuitable for the highly confined and tortuous geometry of the colon. Dehghani et al. (Dehghani et al., 2017) developed a pneumatically driven self-steering colonoscope that could move at an average speed of 28 mm/s and achieve a minimum turning angle of approximately 30°. However, the steering performance relied on continuous pneumatic actuation and a relatively complex soft structure, which may pose challenges for miniaturization and precise control in confined colonic environments. Chen et al. (Chen et al., 2022) designed an inchworm inspired soft robotic colonoscope using rubber bellows, offering high compliance and safety but limited steering accuracy due to pneumatic hysteresis and structural constraints. Vucelic et al. (Vucelic et al., 2006) introduced the Aer-O-Scope system, which enabled full colon examination but retained an operation mode similar to conventional colonoscopy with limited autonomous navigation. As such, while the Aer-O-Scope demonstrates clinical feasibility, its steering capability remains largely operator-dependent, highlighting the need for robotic systems that can provide intrinsic and autonomous directional control. Giri et al. (Giri et al., 2025) developed the *InchIGRAB* mechanism combining guided traction with continuum bending, capable of operating through bends of 80°–130°, though its multi-chamber design increased synchronization complexity and reduced response speed.

Recent advances in compliant continuum robots and snake-like robots have further improved adaptability and manoeuvrability for navigation in confined environments through compliant structures and hyper-redundant locomotion strategies (Liu et al., 2021; Burgner-Kahrs et al., 2015). Although these systems have achieved notable progress, the variable curvature and diameter of the colon, its compliant low-friction wall, and strict limits on allowable contact pressure mean that current approaches remain inadequate in terms of size, control precision, response speed, and the ability to simultaneously achieve reliable propulsion and steering. Consequently, they do not fully satisfy the combined mechanical and control requirements imposed by the colonic environment.

In our earlier work (Du et al., 2025), we demonstrated that a track-based robotic colonoscope with a bellows-driven expansion mechanism could effectively modulate track-wall contact pressure to maintain continuous contact over varying colon diameters and provide the basis for generating steering torque. Building on this foundation, the present study replaces the original bellows-based expansion mechanism with a syringe-driven hydraulic actuation system, enabling controllable track retraction and asymmetric wall contact for force-differential steering.

Compared with representative robotic colonoscope steering strategies, the proposed system provides a favourable balance between steering capability, propulsion performance, mechanical simplicity, and size adaptability. The laboratory-scale prototype successfully negotiates bends of up to 120° while maintaining continuous tracked propulsion, achieves a stable yaw angle of approximately 45° during active steering, and operates over an experimental pipe-diameter range of 95–160 mm. Rather than maximising locomotion speed, the proposed design prioritises steering capability and geometric adaptability, which are critical requirements for negotiating tortuous colonic pathways. Compared with the InchIGRAB mechanism (Giri et al., 2025), which negotiates bends of 80°–130° using a 116 mm front section incorporating a 65 mm pneumatic actuation module with a diameter of 26 mm, the proposed robot achieves comparable bend negotiation through a simpler force-differential steering mechanism integrated into a compact tracked platform without differential track speeds, articulated joints, or external magnetic actuation. This relatively simple architecture enables controllable wall-pressure redistribution while reducing mechanical complexity and maintaining good potential for future miniaturisation.

In this mechanism, selective hydraulic retraction of individual tracks creates an asymmetric distribution of track-wall contact forces, thereby generating a yaw moment while maintaining continuous propulsion. Steering performance is evaluated primarily in terms of planar yaw motion to investigate the feasibility and characteristics of force-differential steering in curved pipes. The following sections describe the operating principle, mathematical modelling, and experimental validation of the proposed approach, and evaluate its steering performance under different geometric and frictional conditions.

## 2 System design and steering mechanism

Compared with the first prototype, the second prototype introduces several improvements in structural design, actuation mechanism, and overall system reliability (Du et al., 2025; Du et al., 2024; Evans et al., 2025). First, the pneumatic bellows based expansion mechanism used in (Du et al., 2025) was replaced with a syringe based hydraulic actuation system. Compared with pneumatic actuation, the hydraulic system uses incompressible liquid for pressure transmission, providing more stable output forces, faster response characteristics, and reduced delay caused by air compressibility. In addition, the original silicone tracks were replaced with TPU (thermoplastic polyurethanes) tracks, improving structural rigidity, wear resistance, and frictional traction against the pipe wall, thereby enhancing propulsion performance and motion stability. Four tracks were selected as a practical compromise between structural simplicity, circumferential support, and steering capability. The symmetric 90° arrangement provides balanced contact around the lumen and allows force-differential steering through independent adjustment of the contact force of each track. Although alternative configurations with different numbers of tracks are possible, their comparative evaluation is beyond the scope of the present study.

**FIGURE 1:**
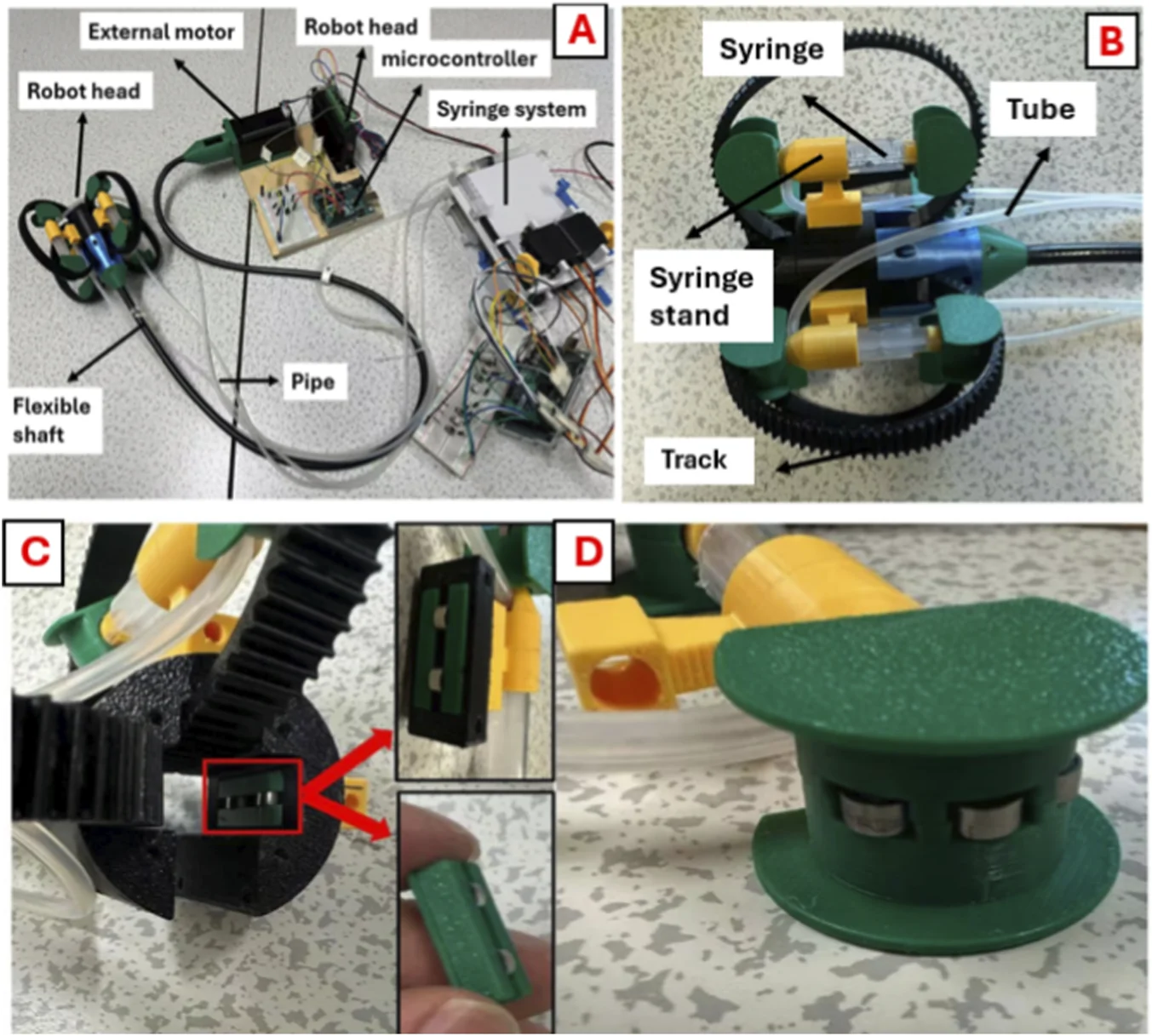
**(A)** The new robotic colonoscope prototype; **(B)** structural configuration of the robot head; **(C)** low friction guiding support structure for track stabilisation. **(D)** modular track support and mounting structure.

From the mechanical perspective, the second prototype further optimised both the transmission and supporting structures. The micro bearings were introduced at both ends of the flexible shaft to improve rotational stability and reduce friction losses during operation, thereby improving torque transmission efficiency and overall system stability as shown in Figure 1. Furthermore, to address the relatively high sliding friction observed between the tracks and the internal supporting structure in the first prototype, a dedicated low friction guiding support structure was designed and integrated into the new prototype system. This structure provides a more stable guiding interface between the inner side of the tracks and the robot body, reducing unnecessary friction and local jamming during track motion, which reduces energy losses and movement jerks.

The track constraint and mounting structures were also further refined. Optimised arc shaped guiding grooves were incorporated into the robot body to limit lateral displacement of the tracks and improve track stability during locomotion. Lubricated contact surface were additionally introduced to further minimise internal friction resistance. To improve maintenance efficiency and experimental repeatability, the second prototype adopts a modular quick release structure, allowing the track modules and expansion mechanisms to be more easily assembled, replaced, and maintained. The track width was also increased from 7.5 mm to 12 mm to further improve the contact area and traction force during locomotion.

These structural and actuation related improvements collectively enhance the propulsion speed, traction and steering stability, enabling more reliable locomotion in complex curved tubular environments.

Figure 1 shows the complete new robotic prototype system, which consists of the robot head, an external motor transmission system, a syringe based hydraulic actuation system, and an electronic control system. The robot head is connected to an external motor through a flexible shaft, allowing the motor torque to be transmitted to an internal worm mechanism that synchronously drives the four tracks. The hydraulic actuation system transfers hydraulic pressure from external syringes to miniature syringes inside the robot, enabling radial deformation of each track module for lumen adaptation and active steering based on differential contact forces. The entire system is coordinated by a microcontroller based control platform, which controls both the propulsion motor and the hydraulic actuation units. This externalised actuation architecture reduces the number and mass of the components that must be accommodated within the robot head, while simplifying internal wiring and improving system modularity and experimental accessibility. However, the current device is a proof-of-concept prototype developed to evaluate the force-differential steering principle and does not represent a clinically miniaturized colonoscope.

### 2.1 Principle of steering mechanism

The proposed robotic colonoscope uses four evenly distributed tracks driven synchronously by a single motor through a flexible shaft and worm gear, ensuring equal linear velocity during propulsion. This has been shown in our previous work (Du et al., 2025). Differential steering is achieved by regulating the normal forces between the tracks and the lumen wall through a syringe driven hydraulic expansion mechanism. Hydraulic input causes each track to expand or retract radially, and selectively retracting one side creates an asymmetric wall pressing distribution that generates a yaw moment. During straight motion the forces remain balanced, while during steering the reduced normal force on one side and the sustained traction on the other induce rotation until the robot realigns with the lumen, after which pressures on the four tracks are equalized. This force differential steering replaces speed differences with controlled variations in wall pressure, and the resulting yaw torque depends on both the magnitude of the normal force imbalance and the effective moment arm. By precisely regulating hydraulic input, the robot maintains stable wall contact and performs smooth, continuous, and reversible steering, enabling reliable navigation through narrow, slippery, and curved lumens.

## 3 Mathematical modelling of the steering mechanism

### 3.1 Geometric constraint analysis

When traveling through a curved section, the robot must satisfy the spatial constraints imposed by the pipe curvature; if its width or length is too large relative to the lumen diameter and curvature radius, it may fail to steer around the bend. A geometric constraint analysis is therefore required to identify the admissible ranges of the width and length that allows smooth passage through curved segments without collision or excessive wall deformation.

**FIGURE 2:**
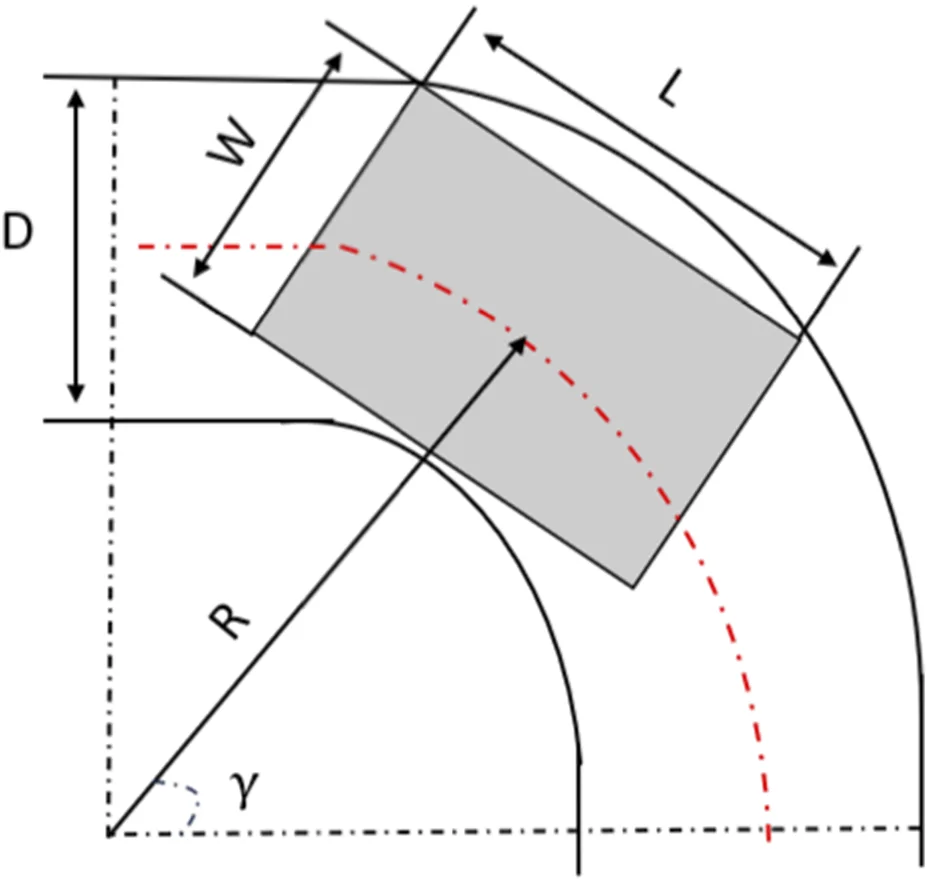
Geometric model approximation of the robot in a curved section.

Figure 2 illustrates the geometric relationship between the robot and the curved pipe, where R denotes the pipe curvature radius, D is the local lumen diameter at the considered cross-section. For simplicity, the local diameter is assumed to remain constant within the geometric analysis, although it varies spatially in the human colon, as shown in Equations 1, 2. W is the robot width, L is the robot length, and γ represents the bending angle of the curved section. Based on this configuration, the following geometric constraints can be derived to describe the feasible range of W and L .

The width, W, of the robot has to satisfy the following relationship:

$$\left(R+\frac{D}{2}\right)\cos\gamma -\left(R-\frac{D}{2}\right)<W<D$$ (1)

To ensure that the robot can pass through pipe section:

$$\frac{L}{2}=\sqrt{{\left(\frac{D}{2}+R\right)}^{2}-{\left(R-\frac{D}{2}+W\right)}^{2}}$$ (2)

### 3.2 Tracks normal force and traction

For simplicity, the friction coefficient between each track and the surrounding wall is assumed to be identical in the present model. In practice, local variations in contact conditions may result in different friction coefficients among the tracks, which could influence the steering behaviour. This effect is beyond the scope of the current model and will be investigated in future work.

**FIGURE 3:**
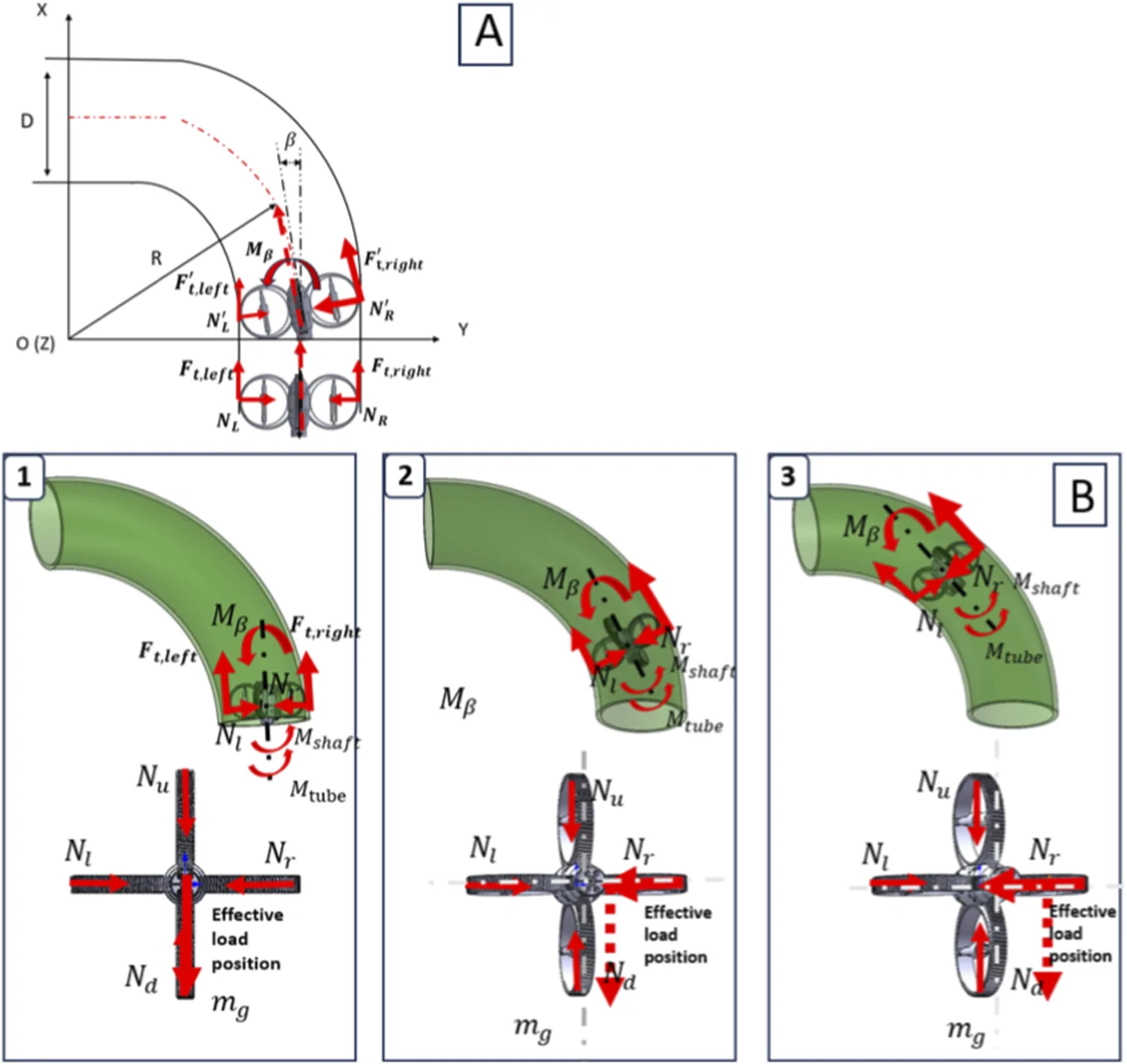
Schematic of the robot steering mechanism in a colon bend: **(A)** 2D geometric and force relationship; **(B)** 3D full-field force and movement analysis.

Considering the robot body as a rigid body with a cross-sectional radius of $r_{b}$ , its yaw (steering) moment originates from the difference in traction between the left and right tracks, expressed in Equation 3:

$$M_{\varphi }=\sum_{i}F_{t,i}r_{b}\sin\left(\theta_{i}-\beta \right)$$ (3)

where β is the yaw angle of the robot relative to the curved pipe coordinate system, as shown in Figure 3.

The terms $N_{l}$ , $N_{r}$ , $N_{u}$ , and $N_{d}$ denote the normal contact forces between the robot tracks and the surrounding wall on the left, right, upper, and lower sides, respectively. These forces are determined by the track expansion state and the redistribution of load due to gravity. As the contact configuration changes, the load is unevenly distributed among the tracks, resulting in variations in the normal forces.

Under the simplified assumption of zero yaw angle:

$$M_{\beta }=r_{b}\left(F_{t,left}-F_{t,right}\right)$$ (4)

At this point, if the right track is retracted (i.e., $r_{right}$ increases, $N_{right}$ decreases), leading to a reduction in the corresponding tangential force. As a result, ${\;M}_{\beta }$ < 0. The steering model described by Equation 4 captures the primary mechanism of yaw generation, which is governed by the traction imbalance between the left and right tracks. Under this formulation, the yaw moment is determined by the difference in tangential forces, assuming a simplified and symmetric distribution of contact forces.

To further illustrate the effect of gravity under varying expansion conditions, Figure 3 shows how the effective gravitational moment changes with track retraction. Under symmetric expansion, the load is approximately evenly distributed and the effective load application point remains close to the cross-sectional centre, resulting in a negligible gravitational contribution to the yaw moment. As the expansion becomes asymmetric, such as when the left track is retracted, the reduced contact on that side causes the load distribution to shift towards the opposite side, moving the effective load application point away from the centre and increasing the moment arm of gravity. Consequently, the effective gravitational moment increases progressively with retraction, as shown in Figures 3B, 1–3. This effect results from changes in load distribution rather than any change in gravity itself. In addition, the overall yaw moment is also influenced by structural effects, including the restoring moment generated by the flexible shaft, $M_{shaft}$ , and the constraint induced moment from the hydraulic tubes, $M_{tube}$ .

Therefore, the yaw behaviour is governed by the combined effects of traction imbalance, gravity-induced load redistribution, and structural constraints, providing a more complete explanation of the observed deviations from the simplified model.

To account for these additional effects, Equation 5 can be extended as:

$$M_{\beta }=r_{b}\left(F_{t,left}-F_{t,right}\right)+M_{shaft}+M_{tube}$$ (5)

Where $M_{shaft}$ , $M_{tube}$ represent the equivalent yaw moments induced by the flexible shaft stiffness and tube constraints, respectively.

The term $M_{shaft}$ originates from the bending and torsional stiffness of the flexible shaft. During steering, deformation of the shaft generates a restoring moment opposing the yaw motion, which can be approximated using an equivalent linear stiffness model, expressed in Equation 6:

$$M_{shaft}=-k_{\theta }\beta$$ (6)

where $k_{\theta }$ is the equivalent rotational stiffness and β is the yaw angle.

The term $M_{tube}$ arises from asymmetric constraint forces induced by the hydraulic tubes. Due to their routing and deformation during steering, the tubes impose uneven forces on the robot body. These forces can be mapped into an equivalent yaw moment using the moment definition, as shown in Equation 7:

$$M_{tube}=r_{b}\left(F_{tube,left}-F_{tube,right}\right)$$ (7)

It should be noted that gravity is not introduced as an independent moment term. Instead, gravitational and structural effects influence the steering behaviour indirectly by modifying the normal force distribution among the tracks, which subsequently alters the available traction forces and resulting yaw moment during configuration changes. The dominant steering mechanism remains the traction imbalance between the tracks, while secondary effects, including the flexible shaft restoring moment and hydraulic tube constraints, provide a more realistic interpretation of the system behaviour and help explain deviations between the simplified model and experimental results, particularly in complex curved environments.

Due to the geometric characteristics of the curved pipe and the influence of friction within the pipe wall, a restoring moment is generated on the robot, pulling its direction back toward the tangential direction of the pipe. This restoring moment acts as a resistive yaw moment against the robot’s steering direction, opposing the turn. In other words, the applied steering moment must overcome the environmental restoring moment to ensure stable steering of the robot, i.e., Equations 8, 9:

$$\left|M_{\beta }\left|\;\geq \;\right|M_{\beta ,f}\right|$$ (8)

$$\left|M_{\beta ,f}\right|=\alpha \mu_{k}N_{total}\frac{r_{b}^{2}}{R_{b}}$$ (9)

where $N_{total}$ is the total normal force on the robot, and α is a calibration coefficient, with $\alpha \in \left(0,1\right]$ .

Accordingly, the steering margin for the robot navigating through the curved pipe can be obtained as Equation 10:

$$\Gamma =\frac{\left|M_{\beta }\right|}{\left|M_{\beta ,f}\right|}\geq 1$$ (10)

where Γ denotes the steering margin.

## 4 Experiment setup and results

### 4.1 Experiment setup

To demonstrate the proposed force-differential steering mechanism and its theoretical model, a series of experiments were conducted on a dedicated test platform to investigate the relationship between hydraulic input and traction, evaluate steering performance in curved paths, and examine the effects of curvature and friction on steering performance. A flexible corrugated pipe was used to provide a compliant and geometrically adjustable test environment for evaluating the steering mechanism. Although this setup provides greater compliance than the rigid acrylic pipes used in preliminary tests, it is not intended to reproduce the full mechanical or physiological characteristics of the human colon. Unlike our previous studies (Du et al., 2025), which primarily employed rigid pipes to evaluate the fundamental locomotion and steering performance of the robot, the present work intentionally adopted a flexible aluminium corrugated pipe to better reproduce the compliant deformation characteristics of the human colon. This experimental configuration enabled a greater focus on evaluating steering performance under more physiologically relevant conditions. Future work will include comparative experiments using media with different stiffness to further investigate the influence of environmental compliance on robot locomotion and steering. Made from lightweight aluminium composite material, the pipe provides both structural flexibility and elastic resilience, allowing it to be manually shaped into various geometries such as single bends (e.g., 90°, 120°) or continuous S-shaped curves while maintaining sufficient restoring force to mimic the passive elasticity of intestinal walls. Its surface friction (enhanced by adding artificial intestinal tissue), slight compressibility, and corrugated texture collectively create more realistic contact and traction conditions than rigid materials. The pipe used in this study had an inner diameter of approximately 152 mm and a length of 1,000 mm, and was fixed onto an acrylic base to ensure stability while allowing curvature adjustments between trials. This flexible environment enabled a more representative evaluation of the robot’s propulsion and steering behaviour under conditions resembling colon endoluminal navigation.

**FIGURE 4:**
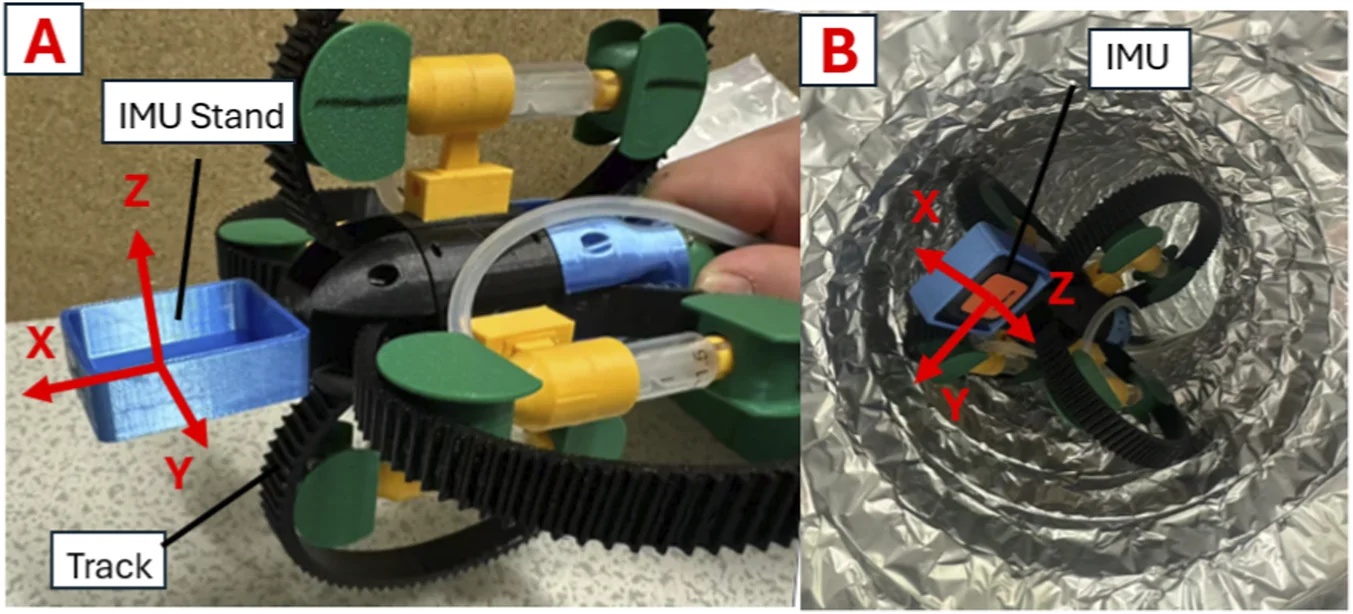
Experimental setup of the robotic colonoscope. **(A)** The fixture designed for mounting the IMU sensor on the robot head. **(B)** The robot during locomotion inside the test pipe, with the X, Y, and Z-axes indicating the IMU coordinate directions (X: forward, Y: lateral, Z: vertical).

The head-mounted IMU (Xsens DOT, Movella, US) accurately reflected robot’s turning motion as it entered and exited curved sections. The sensor was housed within a custom 3D-printed base, which was securely fixed to the front head of the robot to maintain alignment and minimize vibration, as shown in Figure 4. The IMU data were sampled at 15 Hz and recorded through the dedicated software.

For traction measurement, the load cell (DBBSM Load Cell, Applied Measurements Ltd., United Kingdom) was connected to the flexible shaft via a thin cable aligned with the shaft axis. In the propulsion tests, the sensor measured the robot’s axial traction without restricting its motion, while in the normal force tests it was rigidly mounted to the track module to directly capture the wall normal force generated by hydraulic expansion; by incrementally increasing syringe displacement, the quantitative relationship between hydraulic input and normal force was obtained.

### 4.2 Traction force

**FIGURE 5:**
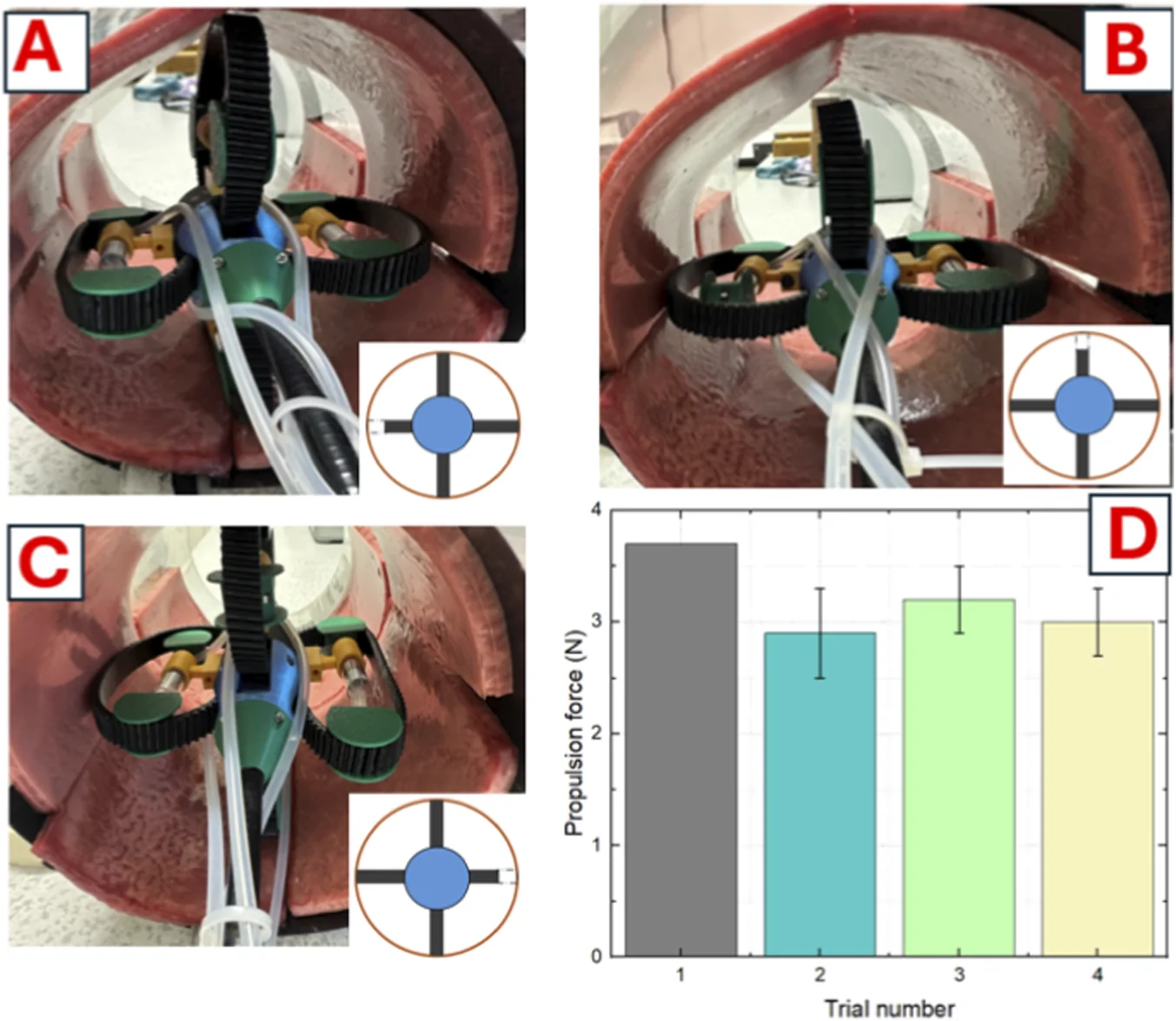
Experimental configurations for traction measurement under different track retraction conditions. **(A)** Left track retracted, **(B)** Top track retracted, and **(C)** Right track retracted, **(D)** Comparison of measured traction forces under different track configurations, where Trial 1 corresponds to the baseline case, and Trials 2–4 correspond to the single-track retraction configurations shown in **(A–C)**.

As shown in Figure 5, retracting one track while the other three maintained a full wall contact created an asymmetric wall-pressure distribution used to examine how localized pressure reduction influences overall traction performance, with the circular insets illustrating the corresponding cross-sectional contact states between the four tracks and the pipe wall.

Retracting one track reduces the total traction due to decreased wall contact pressure. When all four tracks remained in full contact with the wall, the average propulsion force reached 3.7 N, which serves as the baseline performance. When one track was hydraulically retracted, the overall traction force decreased as a result of localized reduction of wall contact pressure. Unless otherwise stated, quantitative experiments were repeated five times, and the results are presented as mean ± standard deviation (n = 5).

For Trial 2, the mean propulsion force dropped to approximately 2.9 N, while Trial 3 produced a slightly higher value of 3.2 N. In Trial 4, the traction was similar to the left-side case, around 3.0 N, corresponding to an approximately 20%–25% reduction relative to the baseline. This trend indicates that retracting a single track substantially reduces the overall driving capability, as the decreased normal force lowers the available traction while the resulting asymmetric wall-pressure distribution generates a yaw moment that diverts part of the traction into rotational motion rather than forward propulsion, consistent with the theoretical predictions. The slightly higher traction observed in Trial 2 is likely influenced by gravity, since the lower track maintained a larger effective normal force due to the contact with the bottom of the pipe during the tabletop experiment. This effect is specific to the current setup and may be less significant in suspended or more clinically representative environments.

**FIGURE 6:**
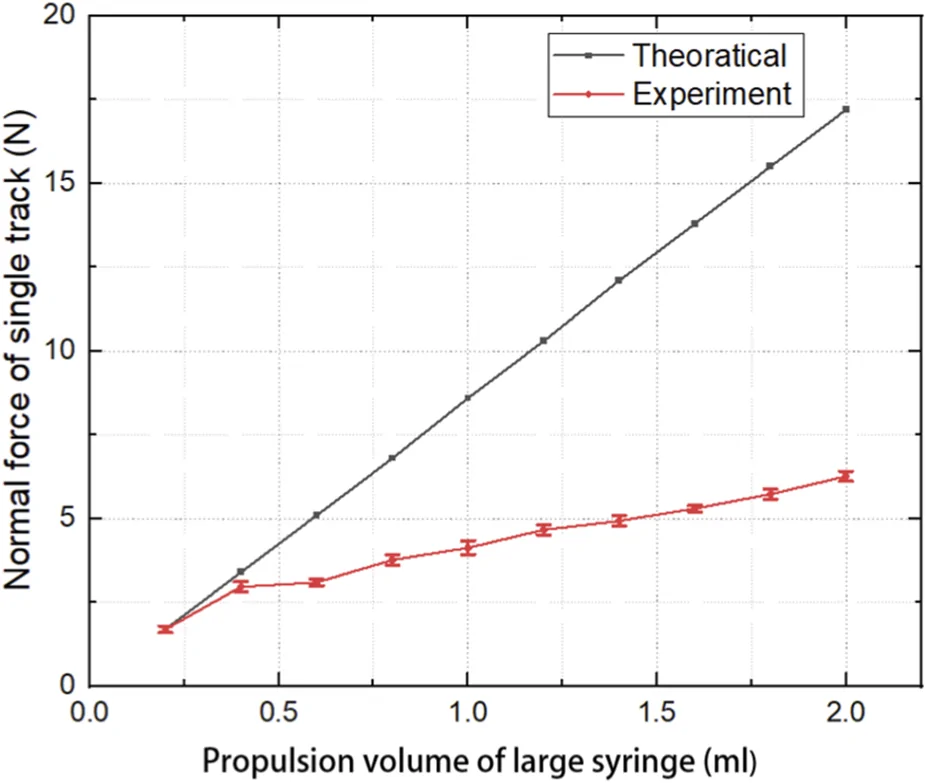
Experimental and theoretical normal forces of a single track under different hydraulic input volumes.

To quantify how the hydraulic actuation input influences the normal force applied to a single track, a load cell (DBBSM Load Cell, Applied Measurements Ltd., United Kingdom) was used to directly measure the wall normal contact force generated during track expansion. The hydraulic input was parameterized by the syringe plunger displacement (or equivalently, injected fluid volume), which serves as a control variable rather than a physical force quantity. During testing, the track module was placed on a rigid support with a load cell mounted behind the support to measure the wall normal reaction force; the large syringe (12 mm diameter) was advanced in 0.2 mL increments, and the small syringe (8.6 mm diameter) transmitted pressure to the expansion chamber, with the steady-state normal force recorded at each step and averaged over five repetitions for the curve in Figure 6.

**FIGURE 7:**
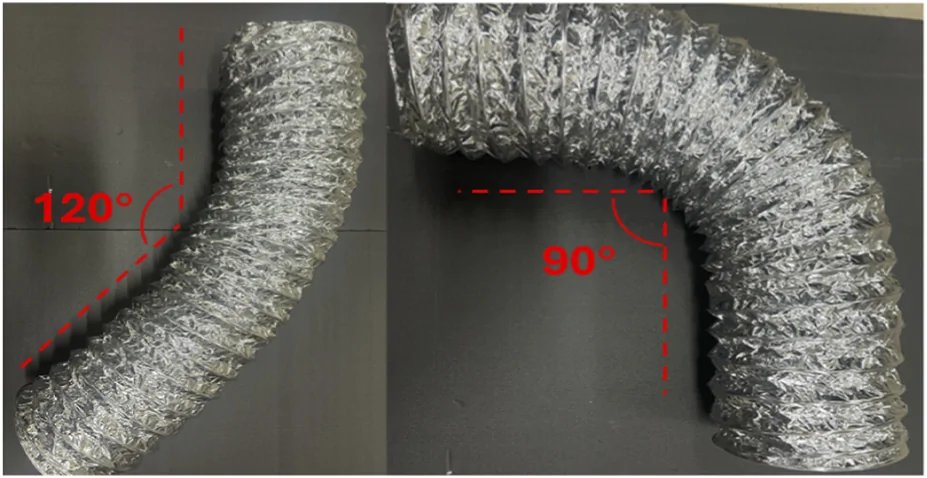
Corrugated aluminium pipe with 90° and 120° bending angles used for steering experiments.

The experimental results (red line) show a near-linear increase in normal force with hydraulic input, but the measured force is substantially lower than the theoretical prediction (black line). At a 2 mL input, the measured normal force was approximately 6 N, compared with a theoretical value of approximately 20 N. This discrepancy is primarily attributed to hydraulic and mechanical losses that are not represented in the simplified analytical model, including fluid leakage, trapped air, tubing compliance, structural deformation, and transmission friction. These non-ideal effects reduce the effective transmission of the input displacement and consequently lower the measured force. Although the simplified model substantially overestimates the absolute force magnitude, it reproduces the qualitative increasing trend observed experimentally. The model should therefore be regarded as a first-order description of the relationship between hydraulic input and track-wall normal force rather than as a quantitatively validated predictor of the physical system. Nevertheless, the experimental results demonstrate that variations in hydraulic input can modulate the track-wall normal force, providing the force-modulation principle required for the proposed force-differential steering mechanism.

### 4.3 Steering experiments

The hydraulic steering system was operated in an open-loop manner during these experiments. Track retraction was manually triggered according to the robot position relative to the bend, without real-time feedback of hydraulic pressure, track-wall normal force, or IMU orientation. Therefore, the timing of hydraulic actuation was determined experimentally and was not automatically compensated for variations in robot speed, slippage, or local contact conditions.

To evaluate steering performance in curved pipes, the Xsens DOT IMU was mounted on the robot head (Figure 4) to provide real-time measurements of yaw rotation about the longitudinal axis, enabling quantitative analysis of planar steering behaviour under different conditions. The experiments examined whether the force differential mechanism can actively generate sufficient yaw motion to assist navigation through bends and investigated the effects of curvature and wall friction on steering behaviour. By comparing active and inactive steering in both single and continuous bends, this section establishes the relationship between hydraulic actuation and the actual steering response recorded by the IMU.

The curved pipes were manually formed from identical flexible corrugated tubing to mimic colon-like geometries, as shown in Figure 7. One bend was mainly used to examine how wall friction affects steering performance, while the other served to compare active and inactive hydraulic steering; both configurations offered realistic curved environments. To assess the performance of differential force actuation steering, the robot was evaluated in the bend under two operating modes. In inactive steering, no hydraulic retraction was activated. All four tracks kept their standard contact height and moved at identical speeds. In active steering, asymmetric track deformation occurred as the robot navigated the bend.

The yaw angle was continuously recorded by the IMU mounted on the robot head. The geometric bending angle of the pipe (120°) corresponds to an actual turning requirement of approximately 60° for the robot, since it moves along the centreline rather than rotating about the curvature centre. The measured yaw angle is slightly smaller than this theoretical value due to the flexibility and deformation of the pipe, as well as the robot’s body vibrations during motion, both of which reduce the effective alignment change detected by the IMU.

Three track-wall contact conditions were used to qualitatively investigate the influence of friction: direct contact with the corrugated pipe surface, a lubricated surface, and contact with artificial intestinal tissue. These conditions are referred to as medium-, low-, and high-friction conditions, respectively, only for relative comparison within the present experiments. The corresponding friction coefficients were not independently measured; therefore, these classifications should be interpreted qualitatively rather than as quantitatively characterised friction levels.

**FIGURE 8:**
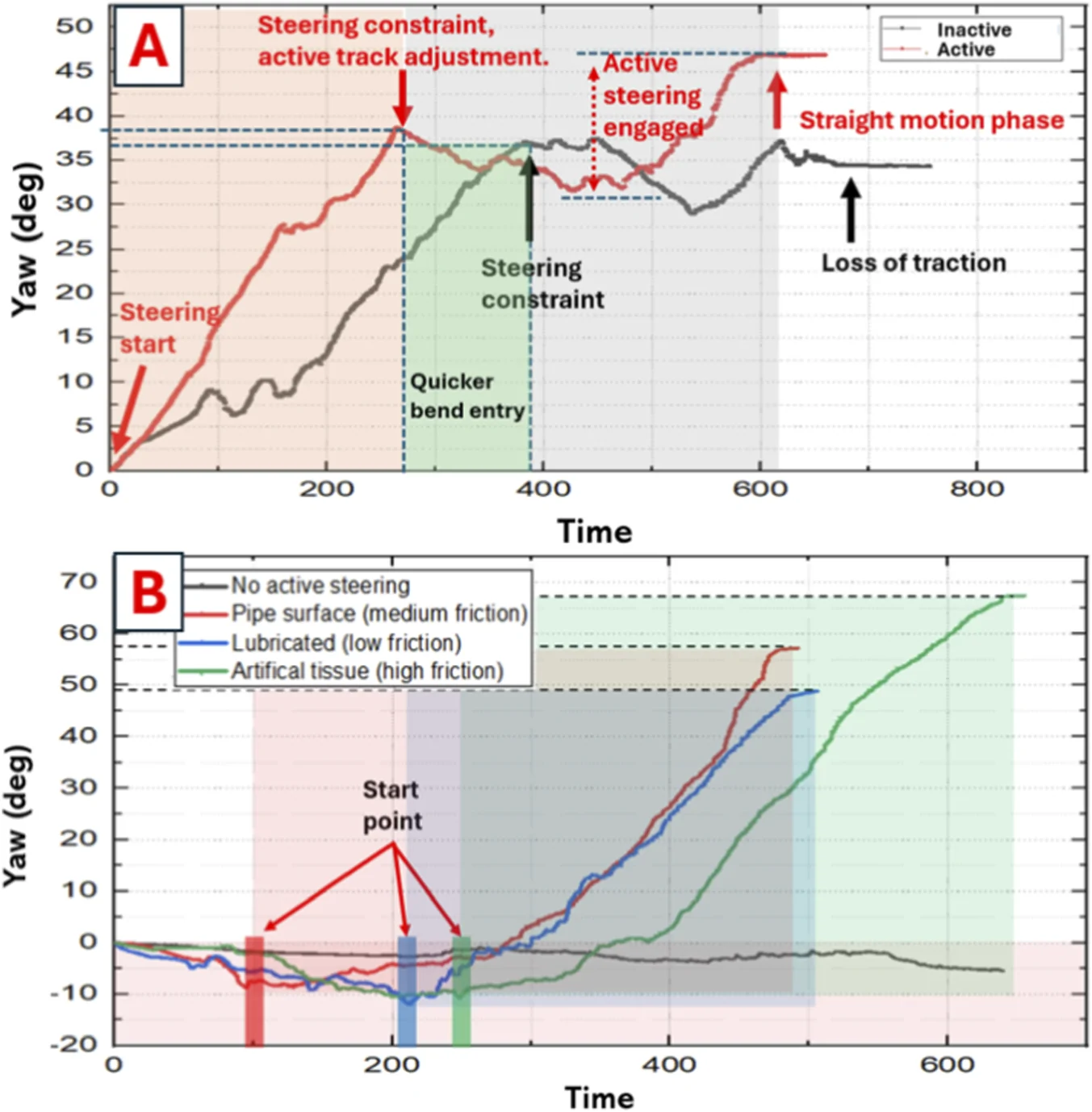
**(A)** Comparison of yaw angle during active and inactive steering in the 120° bend; **(B)** Yaw angle under different wall friction conditions in a 90° bend: pipe surface (medium friction, red), lubricated surface (low friction, blue), and artificial tissue (high friction, green).

As shown in Figure 8A, in the inactive mode, the robot entered the bend with a gradual increase in yaw driven by geometric constraints and frictional interaction with the pipe wall, which saturated at approximately 35°. After about 40 s, the yaw reached a plateau accompanied by fluctuations, indicating traction loss and intermittent track slippage rather than sustained torque generation. In contrast, in the active mode, retracting one side’s tracks deliberately introduced an asymmetric wall-pressure distribution that generated an active yaw moment. This resulted in earlier and smoother entry into the bend, with the yaw angle rising more rapidly to a stable value of approximately 45°. The faster yaw development and higher steady-state yaw demonstrate that force-differential steering provides controlled yaw torque, improves turning performance, and mitigates traction loss, in agreement with the theoretical prediction that hydraulic asymmetry can generate the yaw moment required to negotiate the bend.

At the start of each trial, the robot entered the bend from nearly the same position (indicated by the shaded regions), as shown in Figure 8B). Without active steering, the yaw angle remained around 0°, indicating that passive wall pressure alone could not generate sufficient torque to overcome the curvature. With the force-differential steering, the yaw angle steadily increases, depending on the friction conditions: in the high-friction case (green curve), the yaw angle increased to nearly 80° (from approximately −15°–65° orientation angle),; in the medium-friction case, the yaw increased more slowly and stabilized at 50°; in the low-friction (lubricated) case, yaw angle slowly increased and reached only 40°, where track slipping led to incomplete steering. These results demonstrate that wall friction is critical for effective yaw motion, as higher friction strengthens traction coupling and allows force asymmetry to be transferred into steering torque, whereas excessive lubrication reduces contact force and causes traction loss. Therefore, in low-friction environments resembling the colon mucosa, stable active steering requires either increased hydraulic capability to generate sufficient normal force or a controllable wall-pressure distribution strategy that compensates for reduced friction.

In the second experiment, the pipe is shaped in two consecutive bends −120° bend followed by 135° left bend to simulate complex colon-like curvatures. The start and end positions indicate the robot’s entry and exit points. This geometry was used in IMU-based tests to evaluate the robot’s ability to perform successive steering manoeuvres and maintain traction across consecutive curvature transitions.

Figure 8 primarily compares the achievable steering angles under different friction conditions. The angle-time responses also indicate that higher friction allows effective traction to be maintained for longer, resulting in a more sustained change in orientation. In contrast, under medium- and low-friction conditions, earlier track slippage causes the angular change to decelerate or cease. Because the angular response is non-uniform and affected by intermittent slippage, a single representative angular rate was not extracted in this study. A systematic quantitative analysis of steering rate will be considered in future work. The current prototype employs an open-loop steering strategy and does not include real-time slip detection or compensation. Consequently, track slippage under low-friction conditions may reduce the achievable steering performance. Although the influence of slippage was investigated experimentally under different friction conditions, future work will integrate slip estimation and compensation into a closed-loop control framework to improve steering accuracy and consistency.

**FIGURE 9:**
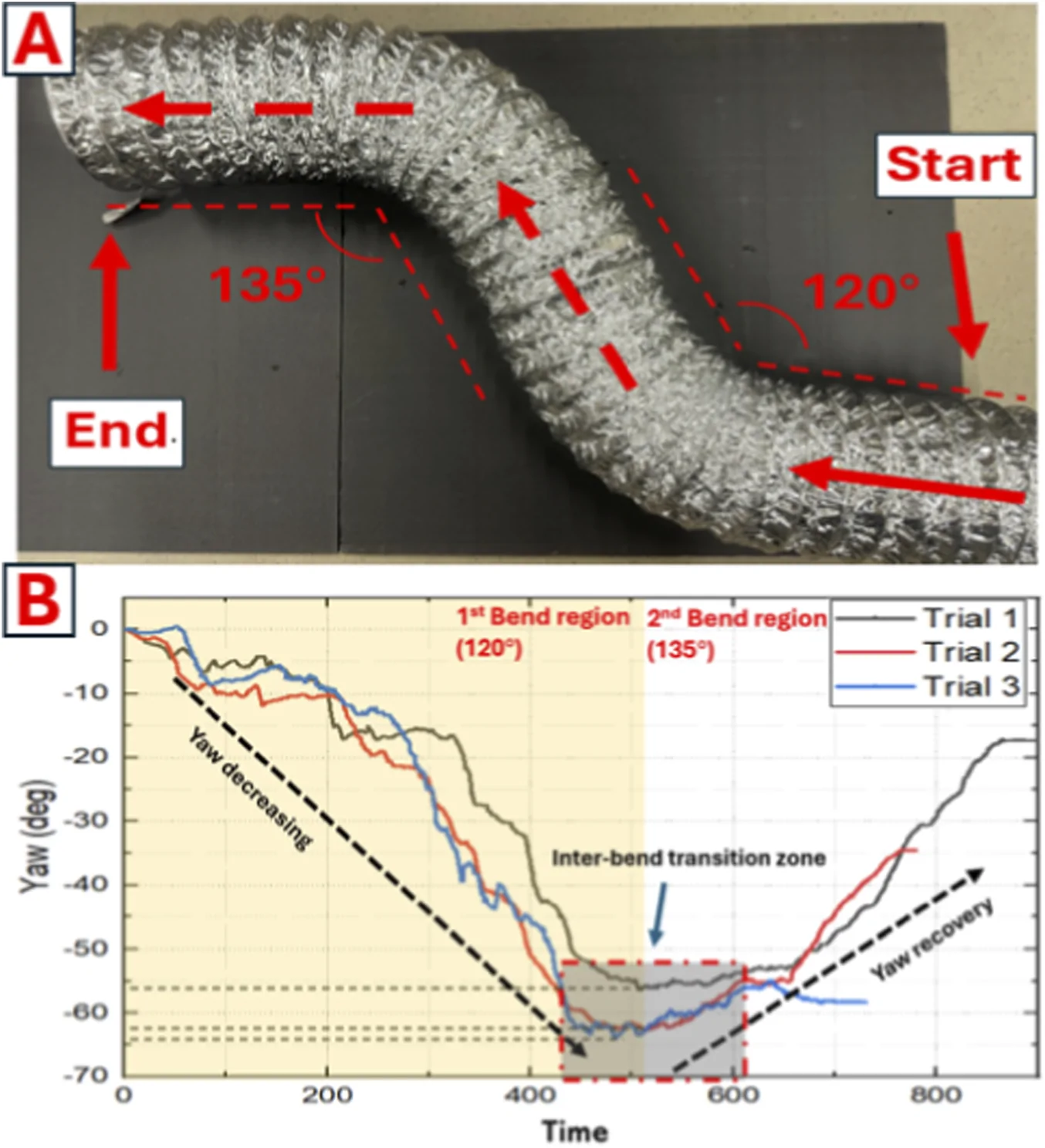
**(A)** Configuration of the continuous S-shaped flexible pipe used for steering experiments, **(B)** Yaw angle change during navigation through a continuous S-shaped pipe.

The corresponding yaw data from three independent trials conducted under the same nominal experimental conditions (n = 3) are shown in Figure 9. All three trials exhibited a similar initial steering pattern, consisting of a yaw-decreasing stage during the first bend and an inter-bend transition region where the yaw angle remained approximately constant at around −60°. Differences became more apparent during the second bend, where yaw recovery depended on the ability of the robot to re-establish the required asymmetric traction.

In Trials 1 and 2, the robot successfully completed both bends. The yaw angle decreased by approximately 55°–65° during the first bend and subsequently recovered during the second bend. Although both trials achieved complete traversal, differences in initial alignment, local contact pressure, friction distribution, and propulsion conditions may have contributed to variations in the timing and rate of yaw evolution. These differences indicate that the steering response was not identical between repeated trials despite the same overall traversal outcome.

In contrast, Trial 3 exhibited incomplete steering after the transition zone. Unlike Trials 1 and 2, the robot did not re-establish sufficient asymmetric traction before entering the second bend. As a result, the yaw angle remained nearly constant and full orientation recovery was not achieved. This behaviour is attributed to the combined effects of local friction variation, pipe elasticity, and the finite response time of the hydraulic actuation system. Variations in local friction affect the traction available for force-differential steering, while pipe deformation can alter the effective track-wall contact conditions. In addition, delayed hydraulic response can reduce the ability of the robot to re-establish the required asymmetric contact force during the transition between successive bends. These interacting effects may therefore account for the incomplete yaw recovery observed in Trial 3 and the trial-to-trial variability across the three experiments.

Overall, two of the three independent trials successfully completed both bends, demonstrating the feasibility of consecutive force-differential steering under the tested laboratory conditions. However, given the limited number of trials and the incomplete steering observed in Trial 3, these results are not sufficient to establish statistical repeatability, robustness, or reliability. Because the four tracks are symmetrically distributed around the robot body and each hydraulic actuation unit shares the same mechanical configuration, the proposed steering principle is not inherently restricted to yaw motion. The yaw experiments presented here therefore serve as a proof-of-concept evaluation of planar steering rather than a demonstration of multi-axis steering capability. Further experiments with a larger number of repeated trials, together with coordinated multi-axis control and evaluation in more physiologically representative environments, will be required to quantify steering repeatability and extend the proposed approach to more complex geometries.

## 5 Discussion and future work

The present study represents a proof-of-concept evaluation of the proposed force-differential steering mechanism rather than validation of a clinically applicable robotic colonoscope. The current enlarged prototype and flexible-pipe environment do not fully reproduce the scale or physiological characteristics of the human colon. Therefore, the reported steering and size-adaptation performance should be interpreted within the tested laboratory conditions. Further miniaturisation and evaluation in more physiologically representative colon models are required before clinical applicability can be assessed.

The experimental results demonstrated that the proposed force differential steering mechanism can effectively regulate traction and orientation in curved pipes, as both force characterization and IMU measurements confirmed that asymmetric traction input produces measurable yaw torque through reduction of wall contact pressure, validating the concept that syringe driven expansion enables steering without differential drive or articulated joints. In the force tests, retracting a single track reduced overall traction by approximately twenty to twenty five percent, and the normal force measurements showed a near linear relationship between syringe displacement and wall pressing force, although the magnitude was lower than theoretical predictions because of pipe deformation and fluid leakage. The approximately linear relationship observed between hydraulic input and normal force is specific to the current prototype and should not be regarded as universally applicable after miniaturization. As the hydraulic components become smaller, trapped air, fluid leakage, tubing compliance, and manufacturing tolerances are expected to have a greater influence on pressure transmission and force generation. These effects may reduce control accuracy and introduce nonlinear behaviour. Future work will therefore focus on improving sealing performance, minimizing trapped air during hydraulic filling, adopting low-compliance tubing, and implementing closed-loop control to compensate for these uncertainties. IMU based experiments further demonstrated the practical performance of the mechanism: in the one hundred twenty degree bend, active steering enabled complete traversal whereas inactive motion stalled; in the ninety degree bend, high friction enhanced traction coupling and yaw response while low friction caused delayed turning; and in the continuous S shaped path the robot completed most trials successfully, with occasional failures caused by insufficient traction.

The three axis acceleration data provided insight into the dynamic interactions among propulsion, wall pressure, and structural vibration. The IMU measurements also contained short-term fluctuations arising from sensor measurement noise in addition to the mechanical vibrations generated during locomotion. However, because the IMU was used for experimental evaluation rather than as a feedback sensor for control, these fluctuations did not affect the interpretation of the overall steering behaviour or the conclusions of the experiments. Despite demonstrating feasibility, the hydraulic system exhibited response delay and hysteresis because part of the input displacement was absorbed by fluid compressibility and tubing compliance, reducing the effective wall pressure and limiting control accuracy. Consequently, although the analytical model correctly captures the increasing relationship between hydraulic input and normal force, it overestimates the absolute force because hydraulic and mechanical losses, including fluid compressibility, tubing deformation, mechanical friction, and transmission losses, were not included in the simplified model. Therefore, the model should be regarded as providing a qualitative prediction of the system behaviour rather than an exact quantitative estimation of the contact force. These effects may become more pronounced during rapid reversals of hydraulic actuation, where the transient response of the hydraulic system can delay the establishment of the desired track-wall contact force, thereby reducing steering responsiveness and force regulation accuracy. Deformation of the flexible pipe introduced additional geometric uncertainty, altering local curvature and contact pressure and contributing to vertical oscillations observed in the FreeAcc-Z signal. The experimental setup also imposed constraints related to scale, speed, sensor stability, and manually operated hydraulic control.

In the present experiments, hydraulic actuation was applied in an open-loop manner, with track retraction triggered at predefined positions rather than through closed-loop pressure or force control. As a result, the effectiveness of steering depended on the relative timing between track retraction and the robot’s progression into the bend. If retraction occurred too early or too late, insufficient force asymmetry was generated at the critical contact region, reducing the available yaw moment and increasing the likelihood of slippage.

A limitation of the current experimental evaluation is that steering was assessed only around a single rotational axis (yaw), which is sufficient for analysing planar navigation in curved pipes but does not capture full three-dimensional orientation dynamics. In the current study, the IMU was used for orientation measurement and experimental evaluation rather than as a feedback sensor for closed-loop control. Consequently, IMU drift did not directly influence the steering control presented in this work. However, for future closed-loop implementations, long-term drift may reduce orientation estimation accuracy during extended operation. This limitation could be mitigated through sensor fusion with additional sensing modalities or periodic recalibration to improve sensing accuracy and support more consistent steering control.

The current system is a proof-of-concept prototype developed to demonstrate the force-differential steering mechanism rather than a clinically sized device. Its external motor and primary hydraulic actuators reduce the number of components required inside the robot head, providing a potential route towards further miniaturization. However, the tracks, hydraulic tubing, internal syringe units, and transmission components still require substantial size reduction. Narrower tracks may reduce the available contact area and traction, while smaller hydraulic components may increase sensitivity to flow resistance, trapped air, leakage, and manufacturing tolerances. In addition, although the rotating components in the current prototype are supported by miniature bearings and the tracks are driven through a worm-gear transmission to minimise internal friction, the relative influence of friction may become more significant after further miniaturisation because of tighter manufacturing tolerances, reduced bearing dimensions, and increased contact stresses. Therefore, the performance of the system at a clinically relevant scale remains to be experimentally demonstrated.

Future work will include multi-axis sensing, interaction force sensing, and closed-loop control to regulate the track-wall contact forces during locomotion and steering, thereby improving steering precision, adaptability, and operational safety, as well as further miniaturization of the track and hydraulic components and evaluation in more physiologically realistic colon models.

## 6 Conclusion

This study presented a proof-of-concept experimental evaluation of a force-differential steering mechanism for a track-based robotic colonoscope. Experimental results showed that asymmetric hydraulic track adjustment can modulate track-wall contact forces and generate yaw motion without differential track speeds or articulated steering joints. The normal-force experiments demonstrated an approximately increasing relationship between hydraulic input and measured contact force, although the simplified analytical model substantially overestimated the absolute force magnitude and should therefore be interpreted as a qualitative description of the actuation behaviour. In curved-pipe experiments, active force-differential steering improved yaw response compared with inactive steering, while the continuous S-shaped experiments resulted in successful traversal in two of three independent trials. These results demonstrate the feasibility of the proposed steering principle under the tested laboratory conditions but do not establish statistical repeatability, robustness, reliability, or clinical performance. Future work will focus on closed-loop force control, compensation for hydraulic response delay and slippage, multi-axis steering evaluation, system miniaturisation, and testing in more physiologically representative colon models.

## Data Availability

The original contributions presented in the study are included in the article/supplementary material, further inquiries can be directed to the corresponding author.
